# Impact of Pectoralis Nerve Block (PECS) on postoperative pain in patients submitted to mastectomy with lymphadenectomy

**DOI:** 10.1590/0100-6991e-20223366-en

**Published:** 2022-11-23

**Authors:** AMANDA LIRA DOS SANTOS LEITE, FREDERICO THEOBALDO RAMOS ROCHA, MICHELLE JACINTHA C. OLIVEIRA, ALDO VIEIRA BARROS, SILVIO MARCOS LIMA DOS SANTOS, ALBERSON MAYLSON RAMOS DA SILVA, DIEGO WINDSON DE ARAÚJO SILVESTRE, ELSON A C FOLHA, CAROLINE C FERRO, TAINA SANTOS BEZERRA, LAERCIO P FACHIN, DALMIR CAVALCANTI SANTOS, CARLOS ALBERTO DE CARVALHO FRAGA, CAROLINNE SALES-MARQUES

**Affiliations:** 1 - Santa Casa de Misericórdia de Maceió, Department of Oncology Surgery - Maceió - AL - Brasil; 2 - Universidade Federal de Alagoas, Programa de Pós Graduação em Ciências Médicas, Faculdade de Medicina - Maceió - AL - Brasil; 3 - Santa Casa de Misericórdia de Maceió, Department of Anesthesiology - Maceió - AL - Brasil; 4 - Centro Universitário Jayme de Altavila - Maceió - AL - Brasil; 5 - Faculdade Pernambucana de Saúde, Department of Estatistics - Recife - PE - Brasil; 6 - Universidade Federal de Alagoas, Instituto de Ciências Biológicas e da Saúde, Programa de Pós Graduação em Ciências da Saúde - Maceió - AL - Brasil

**Keywords:** Breast Neoplasms, Nerve Block, Pain, Postoperative, Pain Measurement, Postoperative Complications, Neoplasias da Mama, Bloqueio Nervoso, Dor Pós-Operatória, Dor, Complicações Pós-Operatórias

## Abstract

**Objective:**

Breast cancer is the most common malignant neoplasm in women worldwide. Surgery has been traditional treatment and, generally, it´s mastectomy with lymphadenectomy, that can causes postoperative pain. Therefore, we seek to study regional anesthesic techniques that can minimize this effect, such as the interpectoral block (PECS).

**Methods:**

*r*andomized controlled study with 82 patients with breast cancer who underwent mastectomy with lymphadenectomy from January 2020 to October 2021 in oncology hospital.

**Interventions:**

two randomized groups (control - exclusive general anesthesia and PECS group - received PECS block with levobupivacaine/ropivacaine and general anesthesia). We applied a questionnaire with Numeric Rating Scale for pain 24h after surgery. We used Shapiro-Wilk, Mann-Whitney and Chi-square tests, and analyzed the data in R version 4.0.0 (ReBEC).

**Results:**

in the PECS group, 50% were pain-free 24h after surgery and in the control group it was 42.86%. The majority who presented pain classified it as mild pain (VAS from 1 to 3) - (42.50%) PECS group and (40.48%) control group (p=0.28). Only 17.50% consumed opioids in the PECS group, similar to the control group with 21.43%. (p=0.65). There was a low rate of complications such as PONV in both groups. In the subgroup analysis, there was no statistical difference between the groups that used levobupivacaine or ropivacaine regarding postoperative pain and opioid consumption.

**Discussion:**

the studied group had a low rate of pain in the postoperative period and it influenced the statistical analysis. There wasn´t difference in postoperative pain in groups.

**Conclusion:**

was not possible to demonstrate better results with the association of the PECS block with total intravenous analgesia. Need further studies to assess the efficacy of the nerve block.

## RESUMO

**Introdução:**

o câncer de mama é a neoplasia maligna mais comum em mulheres no mundo. A cirurgia tem sido o tratamento tradicional e, geralmente consiste em mastectomia com linfadenectomia, podendo causar dor pós-operatória. Por isso, buscamos estudar técnicas anestésicas regionais que possam minimizar esse efeito, como o bloqueio interpeitoral (PEC).

**Métodos:**

estudo controlado randomizado com 82 pacientes com câncer de mama submetidos à mastectomia com linfadenectomia de Janeiro de 2020 a Outubro de 2021, em hospital oncológico. **Intervenções:** dois grupos randomizados (controle - anestesia geral exclusiva e grupo PECS - anestesia geral e bloqueio PEC com levobupivacaína/ropivacaína). Aplicou-se um questionário com Escala Visual Analógica da dor 24h pós-cirurgia. Utilizamos os testes de Shapiro-Wilk, Mann-Whitney e Quiquadrado e analisamos os dados em R versão 4.0.0. Estudo registrado em Ensaios Clínicos Brasileiros (REBec).

**Resultados:**

no grupo PEC, 50% não apresentava dor 24 horas após a cirurgia enquanto no grupo controle, 42,86% negava quadro álgico. A maioria que apresentou dor classificou-a como dor leve (EVA de 1 a 3) - (42,50%) grupo PEC e (40,48%) controle (p=0,28). Apenas 17,50% consumiram opioides no grupo PEC, semelhante ao grupo controle com 21,43%. (p=0,65), (17,50%) grupo PEC e (21,43%) grupo controle (p=0,65). Houve baixo índice de complicações como PONV (náuseas, vômitos, cefaleia) em ambos os grupos. Na análise de subgrupo, não houve diferença estatística entre os grupos que usaram Levobupivacaína ou Ropivacaína quanto a dor pós-operatória e o consumo de opioides.

**Discussão:**

o grupo estudado apresentou baixa taxa de dor no pós-operatório e isso influenciou na análise estatística. Não houve diferença estatística quanto a dor pós-operatória entre grupos.

**Conclusão:**

não foi possível demonstrar melhores resultados com a associação do bloqueio PEC com analgesia intravenosa total. São necessários novos estudos para avaliar a eficácia do bloqueio anestésico no intraoperatório e pós-operatório.

## INTRODUCTION

Breast cancer accounts for 1 in 4 cancers diagnosed among women worldwide^1^. Analyzing the data from the National Cancer Institute (INCA), which estimates new cancer cases for the triennium 2020-2022, an incidence of 66,000 new breast cancer cases in Brazil was observed^2^. In Alagoas, the rate of new breast cancer cases follows the global profile, with a high incidence rate corresponding to 35.20 new cases for every 100,000 women in 2018^3^. 

There are several risk factors associated with the development of breast cancer, being over age 50 as one of the most important factors^2^. There are environmental and behavioral factors such as obesity, alcohol, smoking; reproductive history factors, such as early menarche, nulliparity, late menopause, use of hormonal contraceptives; and genetic factors, such as a family history of ovarian cancer, a family history of male breast cancer, genetic alterations in the BRCA1 and BRCA2 genes^2^ ^,^ ^4^.

The diagnosis of breast cancer is made through a biopsy of the tumor lesion, after a suspected abonormaility is identified on ultrasound or mammography. After that, it is necessary to perform the staging process, which describes aspects of the cancer, such as location and extension, and allows the professional to know the stage of the tumor and define the type of treatment and prognosis of the patient. The 2018 (most current) American Joint Committee on Cancer (AJCC) TNM staging system uses clinical and pathological staging systems for breast cancer^2^.

Early-stage treatment consists of surgery (mastectomy or quadrantectomy) with or without radiation therapy or chemotherapy. Axillary lymph nodes are evaluated by sentinel lymph node biopsy or axillary dissection depending on the initial clinical findings and whether there is metastatic involvement in the pathology. After definitive surgery, adjuvant chemotherapy can be offered to reduce the risk of local and distant recurrence^5^.

Since the late nineteenth century, surgery has been the traditional treatment for breast cancer, and classic radical mastectomy, described by Halsted, remained the treatment of choice for approximately 60 years. In the second half of the 20^th^ century, some changes were introduced in the classic mastectomy, and the techniques that preserved the pectoralis major muscle or both pectoral muscles, described by Patey and Madden, respectively, became known as modified radical mastectomy^6^.

Despite the efficiency of the surgical approach for the treatment of breast cancer, several complications have been reported resulting from these procedures. Among them are lymphedema, surgical wound infection and postoperative pain^7^. 

Patients who underwent mastectomies with axillary dissection suffer postoperative pain and discomfort^8^. Thoracic paravertebral block is the main regional anesthesia technique used in breast surgery, but it does not provide complete analgesia to the anterior and lateral chest wall due to innervations of the supraclavicular, lateral pectoral, medial pectoral and medial brachial cutaneous nerves^8^ ^,^ ^9^. The chronic pain that occurs after axillary dissection is often the result of inadequate treatment of acute postoperative pain^8^. 

Pectoralis I Block (PECS I) was first described by Blanco in 2011. The technique consists of injecting a local anesthetic in the plane between the Pectoralis major muscle and Pectoralis minor muscles, in order to block the medial and lateral pectoral nerves^10^. In 2012, the same author proposed a modified version of the block and it was called the PECS II block, by adding another injection deeper in the plane between the pectoralis minor and the serratus anterior muscle. The technique seems to offer an analgesic advantage for mastectomy and lymphadenectomy, presenting a statistically significant decrease in the Visual Analogue Scale (VAS) pain score and lower analgesic consumption in the postoperative period^9^ ^,^ ^11^.

Postoperative pain in mastectomy patients is known to be intense and requires a large consumption of analgesics, including opioids, which are capable of causing dependence, in addition to causing adverse effects such as nausea, vertigo and constipation^7^. Taking into account the possibility of reducing the consumption through intraoperative anesthetic blocks, as well as the pain control that such a procedure can provide, the objective of this study was to evaluate the effectiveness of PECS anesthetic block in pain control compared to the use of intravenous analgesics in the postoperative period of a mastectomy with lymphadenectomy. Additionally, this study sought to evaluate the average consumption of opioids in 24 hours and the efficacy of the anesthetics Ropivacaine and Levobupivacaine.

## METHODS

### Design and patients

This is a clinical, randomized and controlled trial, carried out at the Santa Casa de Misericórdia de Maceió, Alagoas, from January 2020 to October 2021. The study included women and men with breast cancer undergoing surgery for mastectomy and axillary dissection or sentinel lymph node investigation. Patients with breast cancer who would undergo breast-conserving surgery, drug users, opioid or other routine analgesics users, patients with chronic osteoarticular diseases, fibromyalgia, and bone metastasis were excluded. Those who would undergo breast reconstruction with a latissimus dorsi flap, patients with chronic pain syndromes, allergic to local anesthetics, patients with some contraindication to the use of simple analgesics, local infection at the proposed block site and patients with coagulopathy.

**Figure 1:**
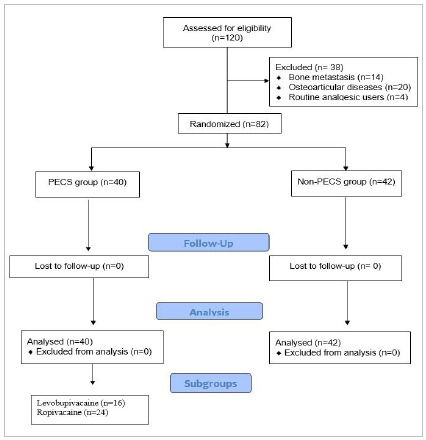
Flow chart of study design, application of inclusion and exclusion criteria, and division of PEC and non-PEC groups.

One hundred and twenty patients with breast cancer who would undergo mastectomy with axillary approach were selected, through the Unified Health System (SUS), in Santa Casa de Maceió. (Figure 1) 38 participants were excluded after applying the inclusion and exclusion criteria (14 had bone metastases, 20 had osteoarticular diseases and 4 were routine analgesics users), leaving n out of 82. The randomization process occurred through a drawing in which some individuals would compose a non-PECS block group and another PECS block group. 

### Ethics

Ethical approval for this study (Ethical Committee N° 3.748.416) was provided by the Ethical Committee of Fundação Educacional Jayme de Altavila/Centro Universitário, Maceió, Alagoas, Brazil (Approved by Ivanilde Miciele da Silva Santos) on 06 December 2019.

### Interventions

The anesthetic used was 0.37% levobupivacaine or 0.3% ropivacaine with a vasoconstrictor with 10ml infiltration between the pectoral muscles and the second puncture of 20ml of local anesthetic between the pectoralis minor and the anterior serratus muscle, guided by ultrasound in the intraoperative phase after general inhalation anesthesia. Sevoflurane and Fentanyl were used for intubation, remifentanil in an infusion pump during surgery. Patients in both groups received a standard analgesic regimen of 1g Dipyrone / Paracetamol, 100mg Tramadol, and 50mg Intravenous Ketoprofen, with morphine rescue as needed. Antiemetics and dexamethasone were also used. No other analgesics were administered during anesthesia in either group. Postoperatively, both groups were prescribed analgesics if necessary, which would be administered in case of pain. 

### Data collection

Data collection was carried out through a questionnaire provided to participants on the first postoperative day, with general information about pathology, pain scale and complications. Participants reported the level of pain they were in and the VAS scale (visual analog scale) was used for this assessment. (0 = no pain, 1-3 = mild pain, 4-6 = moderate pain, and 7-10 = severe pain) (Annex A).

### Statistical analysis

Data description was performed using absolute and relative frequencies, including their respective confidence intervals. Descriptive measures such as the average and standard deviation were calculated. In the statistical analysis of quantitative data, the Shapiro-Wilk test was used to assess the normality of the data, and the Mann-Whitney test to compare the groups. The chi-square test was used to compare proportions. The significance level used was 0.05. Data were consolidated in an Excel spreadsheet and analyzed using R software version 4.0.0.

## RESULTS

A total of 120 eligible patients were selected, 38 were excluded because they did not meet the inclusion criteria, resulting in 82 patients. After applying the criteria, 40 participants were allocated to the PECS group and 42 to the non-PECS group (control). In the analysis of the subgroup of anesthetic use for PECS block, 60% used Ropivacaine and 40% used Levobupivacaine (Figure 1).

Analyzing the variables: gender, comorbidities, histological type of tumor, clinical staging and molecular classification, statistical similarity was observed between the PECS and non-PECS group (Table 1). Females were higher and were present in 95% of the PECS group and 97.6% of the non-PECS group (p=0.563). The main comorbidities found were hypertension (PECS group: 35.90% and non-PECS group: 23.81%) and diabetes mellitus (PECS groups: 7.69% and non-PECS group: 4.76%) (Table 1).

**Table 1:** Distribution of demographic and clinical variables in breast cancer patients in the PECS and non-PECS groups (n = 82).

Variable	PECS group (n=40)	Non-PECS group (n=42)	p-value
	n (%)	n (%)	
Sex			
Male	2 (5.00)	1 (2.40)	0.563
Female	38 (95.00)	41 (97.60)	
Comorbidity			
SAH	14 (35.90)	10 (23.81)	0.448
DM	3 (7.69)	2 (4.76)	
DM + SAH	2 (5.13)	6 (14.29)	
Others	2 (5.13)	1 (2.38)	
No comorbidity	18 (46.15)	24 (54.76)	
Histological Type			
IDC	38 (95.00)	39 (92.86)	0.707
Carcinoma in situ	1 (2.50)	1 (2.38)	0.958
Lobular carcinoma	1 (2.50)	0 (0.00)	0.299
Others	0 (0.00)	2 (4.76)	0.170
Staging			
T1	3 (7.5)	3 (7.14)	0.624
T2	11 (27.50)	9 (21.43)	0.382
T3	13 (32.50)	20 (47.62)	0.759
T4	12 (30.00)	10 (23.81)	0.513
Undetermined	1 (2.50)	0 (0.00)	0.292
Molecular classification of the tumor			
Luminal A	16 (40.0)	12 (28.57)	0.130
Luminal B	13 (32.50)	12 (28.57)	0.097
Triple negative	5 (12.50)	13 (30.95)	0.311
Her2 overexpression	0 (0.00)	2 (4.76)	0.675
Undetermined	6 (15.00)	3 (7.14)	0.352
Neoadjuvant chemotherapy			
Yes	33 (82.50)	34 (80.95)	0.947
No	7 (17.50)	8 (19.05)	0.947

The data are presented as the mean (SD) in each group.*SAH: Systemic arterial hypertension; DM: diabetes mellitus; IDC: invasive ductal carcinoma. **Chi-square test.

Invasive ductal carcinoma was the most present histological type, with 95% in the PECS group and 92.86% in the control group. The TNM clinical oncological staging of the predominant tumor size was T3, present in 32.50% in the PECS group and 47.62% in the control group (p=0.226). In the molecular classification, the luminal subtypes were the most found, 72.5% in the PECS group and 57.14% in the non-PECS group. Most participants underwent neoadjuvant chemotherapy, 82.50% in the PECS group and 80.95% in the control group with p=0.947 (Table 1).

Regarding the type of surgery performed, most patients underwent modified radical mastectomy, 87.5% in the PECS group and 76.19% in the control group (p=0.053) (Table 2). Patients were evaluated during the first 24 hours after surgery regarding the pain index using the VAS scale, opioid consumption and complication rate. The type of surgery performed did not influence the pain scale (Table 3).

**Table 2:** Distribution of types of surgery between the PECS and non-PECS groups.

Variable	PECS group (n= 40)	Non-PECS group (n= 42)	p-value
Modified Radical Mastectomy	35 (87.50%)	32 (76.19%)	0.053
Simple mastectomy + SNLI	3 (7.50%)	10 (23.81%)	
Simple mastectomy + SNLI + Axillary lymphadenectomy	2 (5.0%)	0	

The data are presented as the mean in each group and proportion (%) based in Types of surgery fulfilled. *SNLI: Sentinel lymph node investigation. **Chi-square test.

**Table 3:** Relationship between surgery and the VAS pain scale.

Variable 24-hour post-surgery pain scale	Simple Mastectomy + SNLI	Mastectomy simples + PLS + linfadenectomia	Modified radical mastectomy	p-value
No pain	7 (53.85)	1 (50.00)	30 (44.78)	0.861
Mild pain	6 (46.15)	1 (50.00)	27 (40.30)	
Moderate pain	0 (0.00)	0 (0.00)	9 (13.43)	
Severe pain	0 (0.00)	0 (0.00)	1 (1.49)	

The data are presented as the proportion (%) in each group about relationship between types of surgery and pain scale after 24h postoperative. *SNLI: Sentinel lymph node investigation. **Chi-square test.

**Figure 2:**
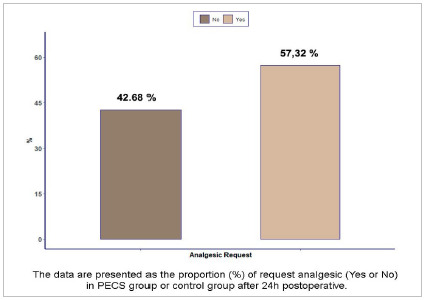
Postoperative analgesic request by breast cancer patients involved in the study.

**Figure 3:**
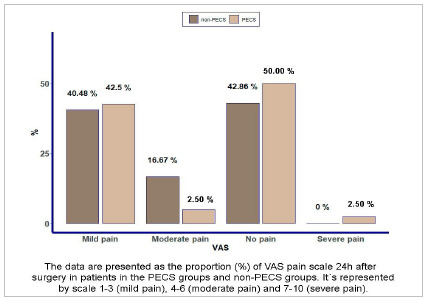
VAS pain scale 24h after surgery in patients in the PECS groups and non-PECS groups.

Regarding the need for postoperative analgesia, most patients required analgesics (57.32%) (Figure 2). Pain data collected in the postoperative 24 hours period were compared between the PECS and non-PECS groups. In the PECS group, 50% of the patients were pain-free 24 hours after surgery while, in the control group, the correspondence was 42.86%. Of those who had postoperative pain, most were classified in the Mild Pain group (VAS from 1 to 3), corresponding to 42.50% in the PECS group and 40.48% in the control group, with p=0.280 (Figure 3). 

Regarding the consumption of opioids, most of the participants did not use the medication (80.49%). In the comparative analysis of the groups, there was a percentage of 17.50% opioid consumption in the PECS group and 21.43% in the control group, a difference that was not statistically significant (p=0.654) (Table 4).

**Table 4:** Postoperative opioid consumption by patients in the PECS and non-PECS groups.

Variable Opioid consumption	PECS group (n = 40)	Non-PECS group (n = 42)	p-value
No	33 (82.50)	33 (78.57)	0.654
Yes	7 (17.50)	9 (21.43)	

The data are presented as the proportion (%) in each group about opioid consumption after 24h postoperative. *Chi-square test.

**Figure 4:**
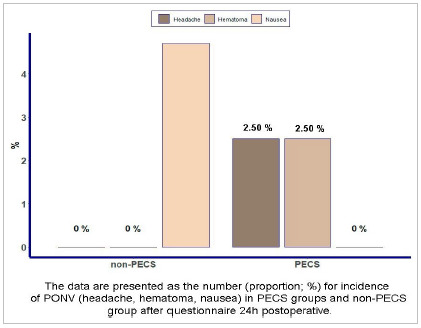
Postoperative complications in patients in the PECS and non--PECS groups.

Regarding the complication rate, a small number was observed in both groups. In the PECS group, no patient had nausea, 1 patient (2.5%) had headache, and 1 patient (2.5%) had hematoma, but without the need for a blood transfusion or surgical re-approach. In the control group, 2 patients (4.7%) had postoperative nausea. There was no statistical difference between the groups (p=0.135) (Figure 4).

Analyzing the PECS group taking into consideration the anesthetic used to perform the block, it is observed that 24 patients (60%) received Ropivacaine and 16 patients (40%) received levobupivacaine. After comparing these subgroups regarding histological type, the tumor staging and the VAS pain scale, it was possible to observe statistical similarity. The most prevalent histological type was invasive ductal carcinoma (93.75% Levobupivacaine subgroup, 91.67% Ropivacaine subgroup, p=0.417). In the T staging, the most frequent were T2 and T3 in both groups. Regarding the pain scale, most patients belonged to the non-pain (43.75% levobupivacaine subgroup, 54.17% ropivacaine subgroup) or mild pain groups (50% levobupivacaine subgroup, 37.50% ropivacaine subgroup, p=0.335) (Table 5).

**Table 5:** Variable analysis in the PECS group according to the types of anesthetic (subgroups).

Variable	lLevobupivacaine	Ropivacaine	p-value
	n (%)	n (%)	
Histological Type			
IDC	15 (93.75)	22 (91.67)	0.417
Carcinoma in situ	0 (0.00)	1 (4.17)	
Lobular carcinoma	1(6.25)	0 (0.00)	
Staging			
T1	0 (0.00)	3 (12.50)	0.327
T2	6 (40.00)	5 (20.83)	
T3	4 (26.67)	9 (37.50)	
T4	5 (33.33)	7 (29.17)	
Molecular classification of the tumor			
Luminal A	7 (43.75)	9 (56.25)	0.035
Luminal B	4 (25.00)	9 (37.50)	
Triple negative	0 (0.00)	5 (20.83)	
Undetermined	5 (31.25)	1 (4.17)	
VAS pain scale			
No pain	7 (43.75)	13 (54.17)	0.335
Mild pain	8 (50.00)	9 (37.50)	
Moderate pain	0 (0.00)	2 (8.33)	
Severe pain	1 (6.25)	0 (0.00)	

The data are presented as the mean (SD) and proportion (%) in each subgroup represented by anesthesia in PECS block with levobupivacaine and ropivacaine. *Chi-square test.

## DISCUSSION

We evaluated the effectiveness of the PECS block combined with intravenous analgesia in controlling postoperative pain after radical mastectomies and simple mastectomies with axillary lymphadenectomy or sentinel lymph node investigation. This anesthetic block did not prove to be superior to the control group in terms of postoperative pain control assessed after 24 hours. The groups were equivalent after analyzing the VAS pain scale. Relatively low pain scores in the first 24 postoperative hours may have made it difficult to detect statistically significant differences in the quality of analgesia in interpectoral block.

A study that evaluated PECS block using three different concentrations of Ropivacaine showed that the peak of pain after modified radical mastectomy was from 24 to 48 hours^12^. Reflecting on the present study, a later analysis could have been performed with the intention of obtaining more reliable responses after applying the VAS scale. 

We suppose that intraoperative analgesia influenced the response to pain 24 hours after surgery. Despite the standardization of medications used in both groups, a study indicated that dexamethasone, commonly used during anesthesia in these patients, not only has an antiemetic effect, but also has great analgesic effects in patients undergoing surgery for breast cancer^13^. This may also explain why did not observed a significant difference in postoperative pain in both groups.

Regarding the consumption of opioids, most of the participants did not use the medication. In the comparative analysis of the PECS and non-PECS groups, there was statistical similarity, with a percentage of 17.50% opioid consumption in the PECS group and 21.43% in the control group. These data are in agreement with the results found in most studies in the literature. A meta-analysis composed of 19 studies analyzed the effectiveness of PECS block after mastectomies, using the 24-hour opioid requirement as the primary outcome and as secondary outcomes, postoperative pain, nausea and vomiting scores. Previous studies revealed results with significantly lower opioid need in the PECS cohort, but reported that the quality of the evidence is low due to heterogeneity and publication bias^14^.

In a meta-analysis published in 2019 consisting of 14 studies, there are 3 clinical trials that did not show statistical significance between the PECS and non-PECS groups with respect to opioid consumption in the first 24h after breast surgery (p=0.07). Furthermore, 11 studies evaluated PECS II block as a cause of significant reduction in postoperative opioid consumption and acute postoperative pain 24 hours after surgery compared to systemic analgesia alone^11^.

Regarding postoperative complications, we observed a small number both in the PECS and non-PECS groups. The percentage of patients who had nausea, vomiting or headache (PONV) in the PECS and non-PECS groups did not show statistical significance. Similar to a clinical trial with 21 patients in the PECS group and 24 in the non-PECS group that brought 17.2% (5 patients) of PONV in the PECS group and 33.3% in the control group^13^. A meta-analysis published in 2020 also investigated the also investigated the incidence of PONV and did not demonstrate a statistically significant difference in its incidence when comparing the PECS block and control groups^15^. These data demonstrate that the PECS block is safe, with low complication rates. 

In a 2019 systematic review, there are also five studies, which included 317 patients, analyzing the impact of the PECS II block on the incidence of PONV. They concluded that there was no significant difference between patients who received the PECS II block and those who received systemic analgesia alone^11^. 

In our study, there were no complications such as pneumothorax or significant bleeding after vascular injury, and we believe that the use of ultrasound has contributed to this. According to Blanco, the ultrasound used to perform the PECS block allows a better identification of the anatomical structures and, consequently, there will be less risk of inadvertent punctures^10^.

Regarding the analysis of the type of anesthetic used for the PECS block, we did not observe any statistical difference between the PECS and non-PECS groups. A study with equimolar doses of the anesthetics levobupivacaine and ropivacaine demonstrated that they exerted similar durations of sensory block (pain) in a model of peripheral nerve block^16^. In another study, comparing analgesia after brachial plexus block using levobupivacaine and ropivacaine, there was also no difference between the groups with respect to postoperative analgesia^17^. Another study found no difference in pain control using 0.25% levobupivacaine, 0.25% ropivacaine and 0.25% bupivacaine^18^. 

This represents the first study, as far as we know, that compares 2 types of anesthetics performed in the interpectoral block in terms of the VAS pain scale and analyzes variables such as histological type of tumor and TNM staging.

As for the cost-effectiveness of the blocking technique, we reached the monetary value of R$ 45.00 per person for performing the PECS, an expense considered low in a large hospital. However, our data do not show benefits in performing the pectoralis nerve block associated with intravenous analgesia, since there were no signs of less postoperative pain and the consumption of opioids was similar between the PECS and non-PECS groups.

We believe that the fact that the studied group had a low rate of pain in the postoperative period influenced the statistical analysis of the groups, and it was not possible to demonstrate better results with the association of the PECS block with total intravenous analgesia. We need further studies to assess the efficacy of the anesthetic nerve block in our patients, with emphasis on intraoperative, immediate and late postoperative periods.

The major limitation of the study was the Coronavirus Pandemic, which started in Alagoas in April 2020, a period in which we had a reduction in the volume of surgeries performed, either due to the death of patients in the preoperative period or due to the lack of beds for cancer patients. Another limitation was the lack of confirmation of the effectiveness of the block before surgery, but we believe that it is more comfortable for patients to perform it after general anesthesia and with less inherent risk to the procedure. In addition, it compromises the blinding of the group, since, when trying to find out if the block worked, it is discovered that the locoregional anesthetic was administered.

Finally, we concluded that there wasn´t additional benefits from the use of PEC block in this group of patients. But, the technique can be tested again in surgeries that cause a higher prevalence of pain.

**CAPTIONS**
AJCC : - American Joint Committee on Cancer
BRCA1 : - Breast cancer 1
BRCA2 : - Breast cancer 2
VAS : - Visual analogue pain scale
PECS : - Interpectoral Anesthetic Block
PONV : - Postoperative nausea and vomiting
SAH : - Systemic Arterial Hypertension
HER2 : - Human Epidermal growth factor Receptor-type 2
DM : - Diabetes Mellitus
IDC : - Invasive Ductal Carcinoma
SLNI : - Sentinel Lymph Node Investigation.
SLN : - Sentinel Lymph Node
TNM : - Classification of Malignant Tumours
MRM : - Modified Radical Mastectomy
